# *QARS1* associated developmental epileptic encephalopathy: first report of a rare homozygous missense variant from Pakistan causing nonepileptic phenotype in a family of seven patients and a comprehensive review of the literature

**DOI:** 10.1007/s11033-025-10574-4

**Published:** 2025-05-31

**Authors:** Riaz Ahmad, Muhammad Naeem, Henry Houlden

**Affiliations:** 1 Medical Genetics Research Laboratory, Department of Biotechnology, Quaid-i-Azam University, Islamabad, 45320 Pakistan; 2 Department of Neuromuscular Disorders, UCL Queen Square Institute of Neurology, Queen Square House, London, WC1N 3BG UK

**Keywords:** Glutaminyl-tRNA synthetase 1, Whole exome sequencing, MSCCA, Progressive microcephaly, Seizures, Cerebral atrophy, Cerebellar atrophy

## Abstract

**Background:**

Pathogenic variants in *QARS1* (MIM:603727; Glutaminyl-TRNA Synthetase 1), which encodes Glutaminyl-tRNA synthetase 1, have been associated with rare progressive microcephaly with seizures and cerebral and cerebellar atrophy (MSCCA MIM:615760). Only a handful of MSCCA patients have been reported in the literature mostly associated with compound heterozygous *QARS1* variants. In the current study, we aimed molecular characterization of a large consanguineous Pakistani family affected with microcephaly, severe intellectual and developmental disability.

**Methods:**

We isolated genomic DNA from blood samples collected from the two affected and three normal individuals of the family. We employed whole exome sequencing, homozygosity mapping, Sanger sequencing and in silico protein modelling tools to characterize the pathogenic variant causing the disease phenotype. Moreover, we collected data of 26 MSCCA patients previously reported in the literature.

**Results:**

The phenotype in the affected individuals of the family was characterized by microcephaly, severe intellectual and developmental disability, but no epilepsy. We found a rare *QARS1* variant NM_005051.3:c.1133G > A, p.(Arg378His) in homozygous state in the family. This variant was recently cited in a patient of Turkish ethnicity, the only MSCCA patient reported with nonepileptic phenotype. This variant lying in the catalytic domain of glutaminyl-tRNA synthetase 1 showed deleterious structure and functional impacts on the protein predicted by in silico tools. The variant was classified as ‘likely pathogenic’ following ACMG guidelines.

**Conclusions:**

We present first report of *QARS1* associated developmental encephalopathy from Pakistan. Our study adds to the restricted clinical and mutational database of this rare disorder supporting the growing evidence that homozygous missense *QARS1* genotypes may lead to the milder phenotype. The reports of more patients with molecular studies will enhance the understanding of the genotype-phenotype correlations.

## Introduction

Progressive microcephaly, seizures, and cerebral and cerebellar atrophy (MSCCA; MIM: 615760) is a neurodegenerative and neurodevelopmental disorder with onset in the first days or months of life. MSCCA is an extremely rare autosomal recessive disease characterized by progressive microcephaly, intractable seizures, cerebral and cerebellar atrophy, moderate to severe developmental delay, and hypotonia. For the first time, Zhang et al. (2014) unpinned MSCCA in 4 patients from 2 unrelated families associated with the *QARS1* gene. In vitro experiments demonstrated a significant impairment of *QARS* aminoacetylation in cell lines, while homozygous loss of QARS function in zebrafish exhibited widespread cell death in the brain with reduced brain and eye size [1]. *QARS1* or glutaminyl-tRNA synthetase 1 (ENST00000306125.12) comprised 24 exons (775 amino acids) with chromosomal position 3p21.31 (https://asia.ensembl.org). So far, few cases of MSCCA have been reported in the literature caused by *QARS1* [1–11]. Animal models including C. elegans, Drosophila melanogaster, Danio rerio and Mus musculus provide insight into the pathogenic mechanism of QARS1-related disorders. Knockdown of QARS-1 through RNAi was generated by Zheng et al. (2022) to assess the functional role of QARS-1 deficiency at cellular and developmental levels [12]. To confirm the role of *QARS1* in neuronal development and structure, Chihara et al. (2007) generated Drosophila melanogaster as a model for the variant of GlnRS (glutaminyl-tRNA synthetase). This study confirmed that the variant had affected the dendritic and axonal development [13]. The loss of function in brain development was confirmed by Zhang et al. (2014) in Danio rerio [1]. *QARS1* knockout was achieved in zebrafish embryos by inserting a gene-trap cassette through retroviral infection.

A large consanguineous family affected with nonepileptic phenotype of MSCCA is presented in this report. Moreover, we summarized clinical and molecular findings of the MSCCA patients harboring the *QARS1* variants previously reported in the literature (Table 1).

**Table 1:** Clinical and genetic data of 28 MSCCA patients identified with *QARS1* variants

Patientss reported	Sex	cDNA and protein change	Zygosity	Prominent symptoms	Age of Onset of epilepsy	MRI SCAN	Ethnicity	Reference
Patient 1	M	c.134G > T, p.(Gly45Val); c.1207 C > T, p.(Arg403Trp)	Compound heterozygous	Severe developmental delay, seizures, pharmacoresistant status epilepticus, microcephaly	1 hour after birth	Enlarged subarachnoid space, dilated lateral ventricles, a thin corpus callosum, atrophy in both the cerebral cortex and cerebellar vermis, decreased contrast of white and gray matter	European American	[1]
Patent 2	M	c.134G > T, p.(Gly45Val); c.1207 C > T, p.(Arg403Trp)	Compound heterozygous	Microcephaly, profound developmental delay, seizures, severe agitation, sensitivity to sound	1st day of life	Enlarged subarachnoid space, dilated lateral ventricles, a thin corpus callosum, atrophy in both the cerebral cortex and cerebellar vermis, decreased contrast of white and gray matter	European American	[1]
Patient 3	M	c.169T > C, p.(Tyr57His); c.1543 C > T, p.(Arg515Trp)	Compound heterozygous	Seizures, profound psychomotor delay, microcephaly, global hypotonia	1hour after birth	Enlarged subarachnoid space, dilated lateral ventricles, a thin corpus callosum, and atrophy in both the cerebral cortex and cerebellar vermis,	European descent	[1]
Patient 4	F	c.169T > C, p.(Tyr57His); c.1543 C > T, p.(Arg515Trp)	Compound heterozygous	Epilepsy, global psychomotor delay, severe hypotonia	1 month	Enlarged subarachnoid space, dilated lateral ventricles, a thin corpus callosum, and atrophy in both the cerebral cortex and cerebellar vermis	European descent	[1]
Patient 5	M	c.1387 C > T, p.(R463*); c.2226G > C, p.(Gln742His)	Compound heterozygous	Small for gestational age, microcephaly, ventricular septal defect, seizures, intractable epilepsy, no psychomotor development	2nd day of life	Profound cerebral atrophy, severely thinned corpus callosum, delayed white matter myelination	Armenian	[2]
Patient 6	M	c.169T > C, p.(Tyr57His); c.1485dup, p.(Lys496*)	Compound heterozygous	Intractable seizures, severe developmental delay, intellectual disability, hypotonia, involuntary movements	4th day of life	Mild cerebral atrophy with left posterior horn enlargement at 7 days; diffuse cerebral atrophy, white matter volume loss, ventriculomegaly, myelination delay at 7 months	Japanese	[3]
Patient 7	M	c.169T > C, p.(Tyr57His); c.1485dup, p.(Lys496*)	Compound heterozygous	Intractable seizures, severe developmental delay, intellectual disability, hypotonia, involuntary movements	1st day of life	Diffuse cerebral atrophy, white matter volume loss, ventriculomegaly, myelination delay, thin corpus callosum at 7 months; progression of ventriculomegaly at 2 years	Japanese	[3]
Patient 8	M	c.1058G > T, p.(Gly353Val) c.1058G > T, p.(Gly353Val)	Homozygous	Cognitive impairment, microcephaly, moderate developmental delay, nystagmus, hypotonia, febrile seizures	Not recorded	Normal brain MRI	United Arab Emirates	[5]
Patient 9	F	c.1426G > A, p.(Val476Ile); c.1426G > A, p.(Val476Ile)	Homozygous	Severe linear growth retardation, microcephaly, facial features, cutaneous syndactyly, high myopia, severe intellectual disability	Infancy	Mild ventriculomegaly	Ashkenazi-Jewish	[4]
Patient 10	F	c.1426G > A, p.(Val476Ile); c.1426G > A, p.(Val476Il e)	Homozygous	Severe intellectual disability, motor delay, high myopia, facial features, and cutaneous syndactyly	Infancy	Ventriculomegaly and hypoplasia of the cerebellar vermis	Ashkenazi-Jewish	[4]
Patient 11	F	c.1426G > A, p.(Val476Ile); c.1426G > A, p.(Val476Ile)	Homozygous	Global developmental delay, hypotonia, failure to thrive, microcephaly, high myopia, mild dysmorphic features, mild to moderate intellectual disability	Infancy	Small periventricular pseudocysts	Ashkenazi-Jewish	[4]
Patient 12	M	c.2084 + 2_2084 + 3 del, p.?; c.793 C > T, p.(Arg25Cys)	Compound heterozygous	Motor development (severely delayed), epileptic encephalopathy, no speech	Not recorded	delayed myelination, hypoplasia and progressive delayed myelination	Not available	[6]
Patient 13	F	c.1430 A > G, p.Tyr477Cys c.1132 C > T, p.Arg378Cys	Compound heterozygous	Infantile spasms, seizures, profound global developmental delay, cortical visual impairment, microcephaly, strabismus	6th week of life	Hypoplasia of corpus callosum, microcephaly	Italian, Korean/Taiwanese	[8]
Patient 14	F	c.1381delC, p.(Gln461Argfs*43); c.199 C > T, p.(Arg67Trp)	Compound heterozygous	Epilepsy, myoclonus, tonic-clonic seizures	1 hour after birth	white matter atrophy, cortical dysplasia, atrophy of vermis cerebelli, enlarged ventricles	European	[7]
Patient 15	F	c.1132 C > T, p.Arg378Cys; c.1567 C > T, p.(Arg523*)	Compound heterozygous	Spasticity at 9 years, epilepsy, focal seizures	1 hour after birth	Hypoplasia of the corpus callosum, progressive atrophy, pachygyria, enlarged ventricles	European	[7]
Patient 16	F	c.1133G > A, p.(Arg378His); c.1133G > A, p.(Arg378His)	Homozygous	Severe developmental delay, no speech	No epilepsy	Hypoplasia of the corpus callosum, white matter atrophy, enlarged ventricles	Turkish	[7]
Patient 17	M	c.1375del, p.(Glu459Asnfs* 45); c.1304 A > G, p.(Tyr435Cys)	Compound heterozygous	Mild developmental delay, epilepsy	Not recorded	Hypoplasia and hypomyelination	Not Available	[7]
Patient 18	F	c.1389–3 C > A; c.134G > T, p.(Gly45Val)	Compound heterozygous	Moderate developmental delay, focal seizures, spasms	5.5 months	moderate atrophy of vermis cerebelli	European	[7]
Patient 19	M	c.2084 + 2_2084 + 3 del; c.793 C > T, p.Arg265Cys	Compound heterozygous	Severe irritability, epilepsy	2.5 months	Hypomyelination, enlarged ventricles, white matter atrophy	Not Available	[7]
Patient 20	F	c.40G > A, p.(Gly14Ser); c.1573 C > T, p.(Arg525Trp)	Compound heterozygous	Severe developmental delay, no speech, epilepsy	2 hours after birth	Hypoplasia of the corpus callosum, pachygyria, enlarged ventricles	American/Hispanic	[7]
Patient 21	F	c.1570 C > T, p.Arg524Trp; c.2068 C > T, p.(Arg690Cys)	Compound heterozygous	Severe developmental delay, epilepsy	3 hours after birth	Hypomyelination, pachygyria, enlarged ventricles, mild atrophy of the cerebral cortex	European	[7]
Patient 22	F	c.170_171insAAA, p.(Tyr57*); c.170 A > G, p.(Tyr57Cys)	Compound heterozygous	Severe developmental delay, dystonia, epilepsy	1 hour after birth	Atrophy of cerebral cortex, pachygyria, enlarged ventricles, delayed myelination	European	[7]
Patient 23	M	c.1207 C > T, p.(Arg403Trp); c.4G > A, p.(Ala2Thr)	Compound heterozygous	Severe developmental delay, myoclonic seizures	4th day of life	Pachygyria, progressive atrophy, enlarged ventricles	European	[7]
Patient 24	F	c.1132 C > T, Arg378Cys c.1574G > A, Arg525Gln	Compound heterozygous	Orofacial dyskinesia, hand stereotypies, focal seizures, developmental delay	6th week of life	Thin corpus callosum, enlarged ventricles, progressive diffuse cerebral atrophy	French-Asian	[9]
Patient 25	F	c.794G > A, p.(Arg265His) c.1485dup, p.(Lys496∗)	Compound heterozygous	Global moderate developmental delay, progressive microcephaly, seizures	At birth	Macrogyria, leukoaraiosis, decreased density of white matter in bilateral frontal, temporal, and parietal lobes, Extensive edema, shallow and sparse cerebral sulcus indicating possible macrogyria	China	[10]
Patient 26	M	c.794G > A, p.(Arg265His) c.2234G > A, p.(Arg745His)	Compound heterozygous	Severe pharmacoresistant seizures, progressive microcephaly, mixed hypotonia and hypertonia, minor facial dysmorphia, cortical visual impairment	At birth	Moderate supratentorial cortico-subcortical atrophy, enlarged ventricular system, massive corpus callosum atrophy, diffuse cortico-subcortical atrophy with T2 hyperintense white matter and thinning of the cortical ribbon	Tunisian	[11]
**Patient** **27 (V:5)**	M	c.1133G > A, p.Arg378His c.1133G > A, p.Arg378His	Homozygous	Severe intellectual disability, mild growth delay, slurry speech, no epilepsy	No epilepsy	Normal brain MRI	Pakistani	**Current study**
**Patient** **28 (V:7)**	M	c.1133G > A, p.Arg378His c.1133G > A, p.Arg378His	Homozygous	Severe intellectual disability, mild growth delay, slurry speech, no epilepsy	No epilepsy	Normal brain MRI	Pakistani	**Current study**

## Materials and methods

### Ascertainment of patients

All experiments involving human subjects or related data were conducted in accordance with relevant ethical regulations. Informed consent was signed by the affected family members and ethical approval for this study was obtained from the Institutional Review Board (IRB) of Quaid-I-Azam University, Islamabad. Detailed clinical history and venous blood samples were collected from the available healthy and affected individuals at the time of ascertainment. Peers of the affected family were interviewed to construct pedigree and collect disease history. Through standard protocol, DNA was extracted from peripheral blood lymphocytes.

### Genetic analysis by next-generation sequencing

Whole exome sequencing for the subject V:5 was performed at Macrogen (Korea) by using the Agilent SureSelect Human All Exome V6 Kit (Agilent Technologies, Santa Clara, CA, USA) as described [14]. High-throughput sequencing system, Illumina NovaSeq 6000 (Illumina, Santa Clara, CA, USA) was used for paired-end sequencing or PE150. The obtained sequencing reads were aligned to the reference genome via Burrows-Wheeler Aligner v0.7.17 (BWA; http://bio-bwa.sourceforge.net/). For the enrolled family, assembly hg19 was selected, while hg38 genome assembly was utilized for the sequencing reads. BAM files were sorted through SAMtools (v1.8) while duplicated reads were identified with the help of Picard (v2.18.9). Genotyping was conducted through the Genome Analysis Toolkit (GATK; http://www.broadinstitute.org/gatk/) v4.0. Functional annotation and filtration of variants were performed using ANNOVAR (http://www.openbioinformatics.org/annovar/) and FILTUS, respectively. After annotation, the obtained file was saved in CSV format and filtered to find the most likely pathogenic variants. Our filtration strategy concentrated on exploring the coding regions as well as the splice acceptor and donor site. Based on the clinical history and pedigree, we chose the rarest variants with minor allele frequency (MAF < 0.01%) in public databases including the Genome Aggregation Database (https://gnomad.broadinstitute.org/) http://evs.gs.washington.edu) and 1000 Genomes project (http://www.1000genomes.org/). Different in silico tools were also used to predict the functional effect of the filtered variants. According to the ACMG guidelines, the identified variants were classified aspathogenic, likely pathogenic or variant of uncertain significance (VUS).

### Homozygosity mapping and in Silico tools

**Fig. 1:**
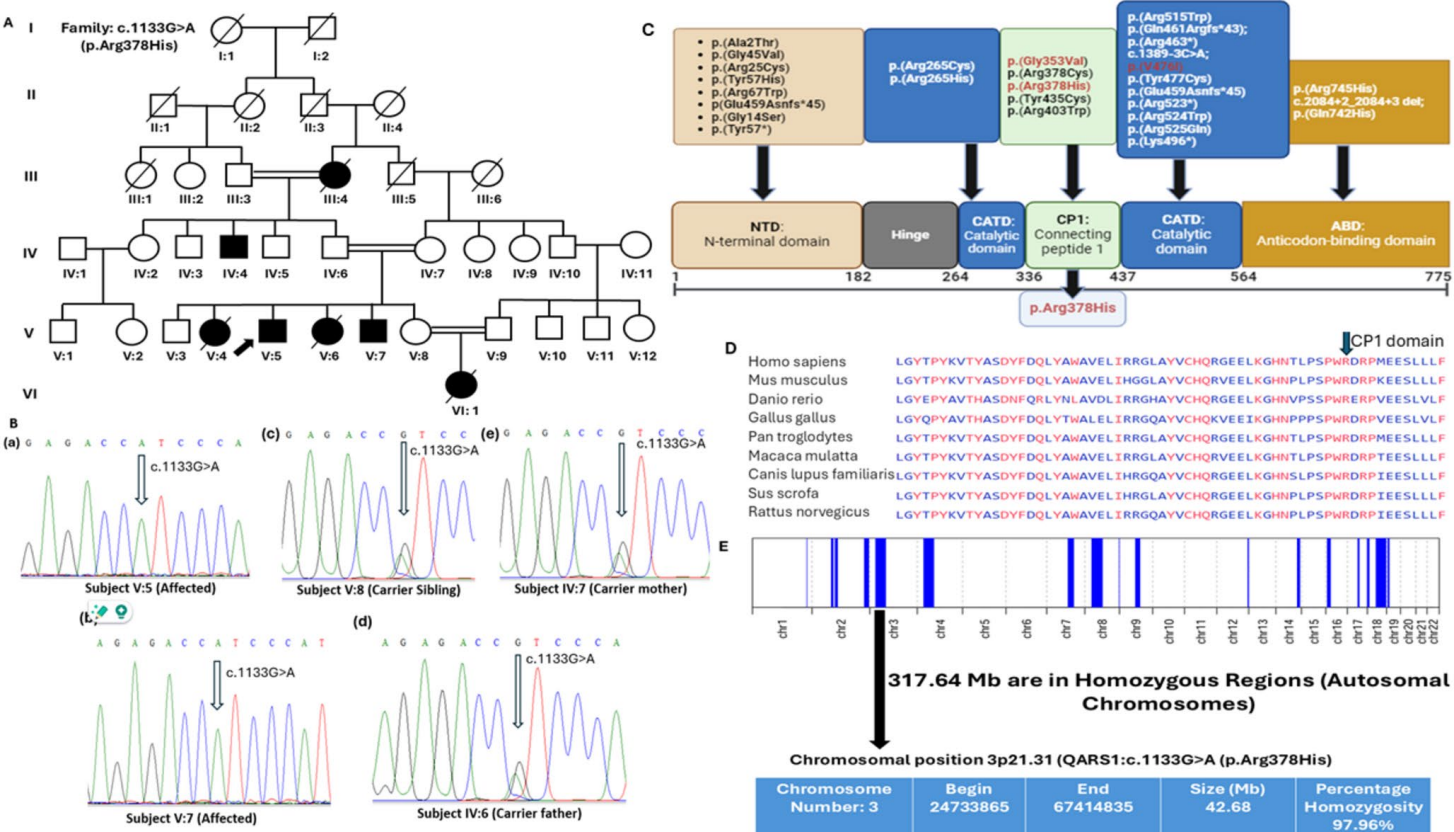
(**A**) Pedigree of the consanguineous Pakistani family affected with MSCCA. (**B**) Sanger sequencing of the healthy and affected individuals. (**C**) Domains and reported mutations of QARS1 protein. (**D**) Evolutionary conservation of the mutated QARS1 residue p.Arg378. (**E**) Homozygosity mapping using AutoMap tool

AutoMap (https://automap.iob.ch/process) tool with the default settings on the VCF (Variant Call Format) file of whole exome sequencing was used for homozygosity mapping (shown in Fig. 1) because the identified variant was found in the region shared by ancestors [15]. The pathogenicity of our selected variant was confirmed by PolyPhen-2 (http://genetics.bwh.harvard.edu/pph2), MutationTaster (www.mutationtaster.org), SIFT (http://sift.jcvi.org/), CADD (Combined Annotation Dependent Depletion (https://cadd.gs.washington.edu), DANN, VarSome (https://varsome.com/), PROVEAN, EIGEN and VARITY. The detected variant was further classified) based on ACMG guidelines through the Franklin variant assessment tool (https://franklin.genoox.com/clinical-db/home).

### Sanger sequencing validation

To validate c.1133G > A, p.(Arg378His) in the *QARS1*, we amplified genomic DNA of the affected and normal individuals of the family (Fig. 1) with a pair of primers (Forward: CCAGGGCCATGACGTAGAG and Reverse: CCAAACCCAGATGCTCCTCT) and sequenced with Sanger method by forward primer. These primers were designed with the Primer3 tool (https://primer3.ut.ee/). Although NGS has considerably improved accuracy, it is usually recognized that the identified variant be validated with Sanger sequencing prior to reporting [16]. To date, using whole-exome sequencing, 188 variants in 143 distinct genes have been reported in the Pakistani population as described previously [17].

## Results

### *QARS1* biallelic variant and algorithms

Whole exome sequencing in our enrolled family was used to identify a single genetic variant that fulfilled the criteria for causality based on established protocols [18]. Family had homozygous *QARS1*: NM_005051.3: c.(1133G > A) p.(Arg378His) variant. The candidate variant was validated by Sanger sequencing amongst family members following the recessive mode of inheritance (Fig. 1). Minor allele frequencies; exomes: ƒ = 0.0000137 (cov: 57.9) genomes: ƒ = 0.00000657 were available in VarSome (https://varsome.com/). Algorithms such as SIFT (deleterious (0), Polyphen2 (probably damaging (0.996), MutationTaster (disease-causing), DANN (deleterious (1), PROVEAN (pathogenic supporting), EIGEN (pathogenic supporting (0.7135), MutPred (pathogenic supporting (0.671) and VARITY (deleterious (0.94) predicted our variant as disease-causing or deleterious. Furthermore, the CADD score for the identified variant was also high (28.4), indicating a pivotal role in the pathogenesis of the disease.

### Biallelic variant associated with MSCCA phenotypes

Our family comprised of seven patients (III:4, IV:4, V:4, V:5, V:6, V:7 and VI:1) as shown in Fig. 1. Two of them, V:5 (15 years old) and V:7 (12 years old) were born without pregnancy complications. Symptoms of both were consistent with MSCCA, except for lack of epilepsy. Their clinical features were microcephaly, poor weight gain, severe intellectual disabilities, and slurry speech. They also exhibited symptoms such as mild facial dysmorphia including a sloping forehead, broad flat nasal bridge and deep-set eyes. Other features were mood instabilities, anxiety reactions, learning disabilities (writing and reading), less responsiveness to tasks or questions, short-term memory, poor judgment or decision-making ability and poor communication to maintaining or initiating conversations. Symptoms of the affected individual III:4 were mild alive at 90 years of age, while his affected son (IV:4) died at 50 years of age. The affected individual V:4 had moderate symptoms and died at the age of 5 years due to complications from measles, while the affected individual V:6 died at the age of 8 years following an accident. Biochemical, metabolic and karyotyping evaluations were normal in the proband (V:5). Retinal examination, hearing tests, electroencephalogram, and brain MRI scans were normal for the affected individuals (V:5 and V:7).

### *QARS1* variant alters evolutionarily conserved amino acids

Our reference amino acid (arginine at 378 position) is highly conserved among different species (Homo sapiens, Mus musculus, Danio rerio, Gallus gallus, Pan troglodytes, Macaca mulatta, Canis lupus familiaris, Sus scrofa, Rattus norvegicus) as presented in Fig. 1. Functionally confirmed variants are highly conserved as examined by Zhang et al. (2014) [1]. Therefore, conservation across diverse taxa highlights the evolutionary importance of our mutated catalytic domain, indicating further investigation into its functional role in related pathologies.

### Impact of variant on protein function or structure

**Fig. 2:**
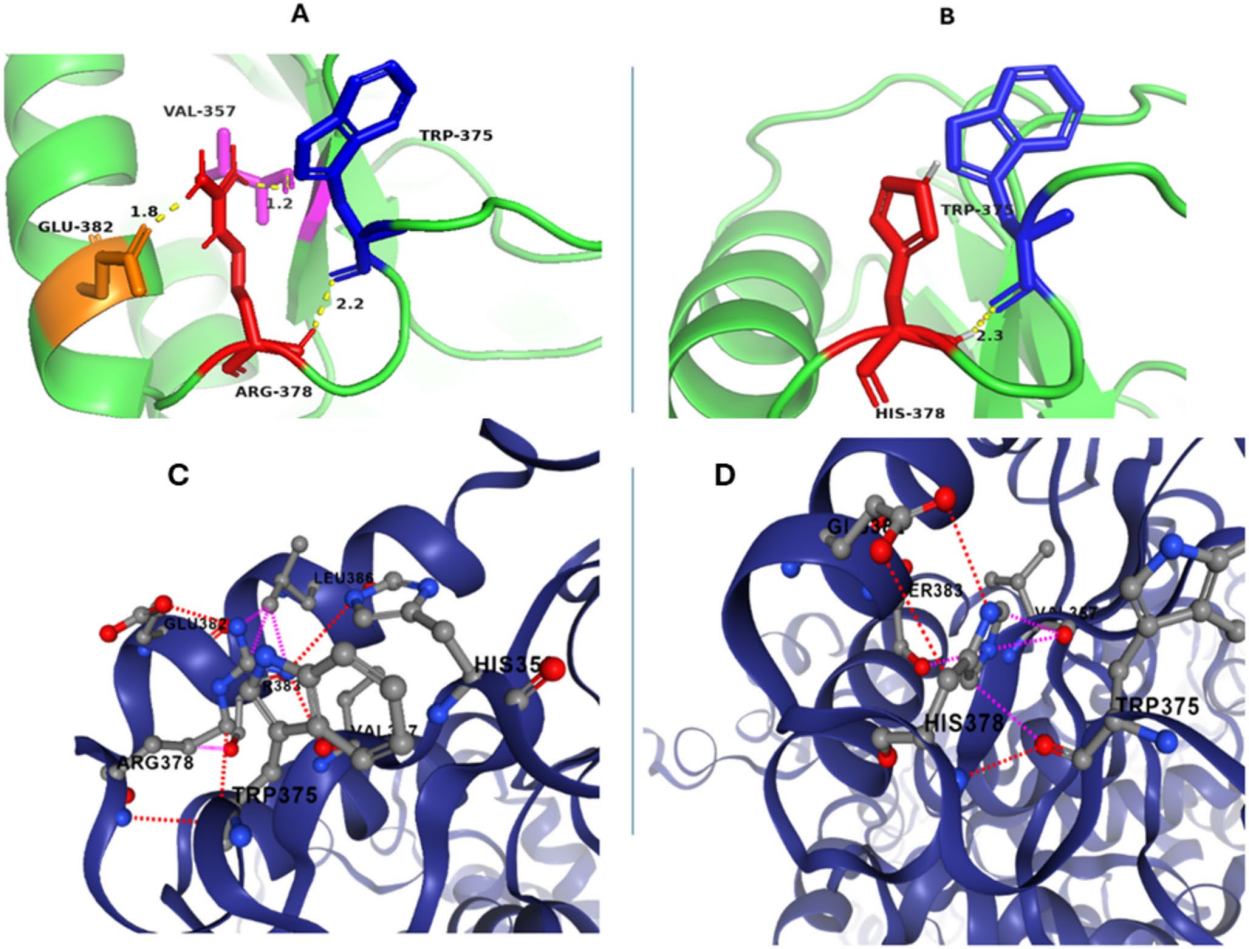
(**A**). Wild-type QARS1 protein where Arg378 (red) makes H-bonds with Trp375 (blue) 2.2Å, Val357 (magenta) 1.2 Å and Glu382 (orange) 1.8 Å. (**B)**. Mutant QARS1 with His378 (red) depicts H-bond with Trp375 (blue) 2.3 Å. (**C**). Wild-type protein with H-bonds interactions of Arg378 with neighboring amino acids where dotted red lines depict H-bonds. **D.** Mutant protein His378 where red dotted lines show the H-bonds and purple dotted lines depict the protein structure clashes in both (**C**) and (**D**)

Protein modeling of the mutated *QARS1*: p.Arg378His was done by the SWISS Model (https://swissmodel.expasy.org, accessed on 14th October 2024), visualized with Pymol (https://www.pymol.org, accessed on 14th October 2024) and compared with the wild -type protein present in AlphaFold 3 database (https://alphafold.ebi.ac.uk/entry/P47897, accessed on 14th October 2024). Comparison depicts that Agr378 makes H-bonds with Trp375, Val357 and Glu382 whilst the mutant His378 makes H-bond with Trp375 only (Fig. 2). Both Arg and His are polar amino acids and positively charged but the imidazole ring in His indicates weak and conditional charge in both protonated and deprotonated forms which can contribute to less bonding (Fig. 2B) and protein structure instability. Mutational analysis by the DynaMut (https://biosig.lab.uq.edu.au/dynamut/, accessed on 14th October 2024) also suggests destabilization of protein with Δ ΔG ^stability^ = -1.24 kcal/mol score. Figure 2 indicates that Arg378 (wild) has a guanidinium side chain that favors interactions and bonding, stabilizes the structure and contains clashes with Leu386 and Ser383 (purple dotted lines in Fig. 2). On the other hand, His378 makes steric hindrance with Trp375, Val 357 and Ser383 which is not present in wild-type protein. The size difference in both Arg378 and His378 further provides evidence of instability of the protein’s active site conformation, making it unable to precisely bind with the tRNA.

## Discussion

So far, approximately 28 MSCCA patients have been reported in the literature caused by *QARS1* including the two patients in our conducted study. Only five patients are reported in homozygous condition and the rest are compound heterozygotes. This report presents the study of Pakistani family affected with MSCCA caused by a missense variant NM_005051.3:c.1133G > A, p.(Arg378His)classified as likely pathogenic (PM2, PM5, PP1, PP4, PM3). Clinical findings were progressive microcephaly, poor weight gain, severe intellectual disabilities, minor facial dysmorphia, and slurry speech. The 3D analysis of both mutant and wild-type GlnRS protein revealed potential structural changes induced by the homozygous mutation in the catalytic domain (Fig. 1).

MSCCA patients generally exhibit a triad of symptoms such as primary or secondary microcephaly, epilepsy with neonatal or infantile-onset and moderate to severe intellectual disabilities as shown in Table 1. Our patients are extremely rare in the literature presenting with non-epileptic symptoms [5, 7], slurry speech and normal brain MRI while the single female patient reported with the same homozygous mutation NM_005051.3:c.1133G > A, p.(Arg378His) [7] had no speech and an abnormal MRI scan (hypoplasia of the corpus callosum, white matter atrophy and enlarged ventricles).

Most of the patients with homozygous variants are reported with milder phenotypes [5, 7]. indicating homozygosity might be protective for the risk of developing epilepsy [7]. The same is the case in our patients in the context of homozygosity and milder phenotype. In contrast, compound heterozygous mutations showed severe phenotypic manifestations, with some exceptions. Two patients were mildly affected by the compound heterozygous mutation, possibly because of null and hypomorphic allele phenomena [7].

Anxiety and mood instabilities were seen in a patient reported by Zhang et al. (2014), who had sensitivity to sound and severe agitation [1]. These symptoms are consistent with our proband (V:5) phenotype. Craniofacial anomalies including facial dysmorphia were also reported by Leshinsky-Silver et al. (2017) [4], Chan et al. (2022) [9] and Sakka et al. (2024) [11] and matched with all patients of our family. The most common feature of severe intellectual disability (ID) was noticed in different studies [1, 3, 4, 7].

## Conclusions

Our study diagnosed a familial case consistent with MSCCA, enabled by whole exome sequencing. Notably, just a single patient of Turkish ethnicity was already cited but our study further explores the spectrum in the context of disease severity, zygosity and CP1 domain of glutaminyl-tRNA synthetase 1. Our study supports the potential role of the variant in the mild form of MSCCA because our patients and a single patient of Turkish ethnicity are mildly affected without epilepsy. Functional studies should be encouraged in MSCCA patients exhibiting *QARS1* variants, for insights into exploring the disease pathogenesis.

## Data Availability

No datasets were generated or analysed during the current study.
